# 636. Access to Mental Health Services at Burn Centers in the U.S.

**DOI:** 10.1093/jbcr/irag033.462

**Published:** 2026-04-06

**Authors:** Diana B Griffin, Robert Galiano

**Affiliations:** Northwestern University, Chicago, IL; Northwestern University, Chicago, IL

## Abstract

**Introduction:**

Burn injury survivorship is multidimensional – outcomes depend on injury severity, surgical management, and psychosocial adaptation. The study aims are trifold: 1. characterize mental health services at U.S. burn centers; 2. quantify regional mental health burden in the general population as a proxy for pre-burn status; 3. compare aim one and two findings to identify service shortfalls.

**Methods:**

Using the American Burn Association (ABA) directory, a list of burn centers was developed. A comprehensive search was conducted across all burn center domains and affiliated websites by scraping and manual verification. This gathered burn center specific information including location, ABA verification status, populations served, and mental health services offered. SAMHSA’s National Survey on Drug Use and Health supplied state-level estimates of any mental illness and treatment in the prior year. Data analysis contextualized trends for mental health need and resource availability.

**Results:**

The ABA directory contains 126 U.S. burn centers, 89 ABA-verified (56 adults, 17 pediatric, 16 both). Burn center distribution is uneven: 9 states with no burn centers, 18 with one, 6 with two, 9 with three, and 9 with four or more. There exists a strong correlation between state-level number of burn centers and population (r = 0.907, *p*<1e-18). Among states with at least one burn center, mental illness prevalence positively correlates to adult burn centers per capita (r = 0.41, *p*=.02). However, after excluding the high influence outlier (Washington D.C.), the correlation weakens (r = 0.234, *p*=.214). Adult burn centers per capita positively correlate with mental health treatment in the last year (r = 0.483, *p*=.0059). Quantity of mental health resources at burn centers shows a weak inverse relationship with state mental health illness prevalence (r = -0.238, *p*=.0925).

**Conclusions:**

This study demonstrates that the placement of burn centers is primarily population-driven, not tuned to mental-health burden. Although a mild relationship exists between mental health need and the presence of adult-servicing burn centers, more can be done to efficiently plan and allocate resources to target areas with larger unmet need.

**Applicability of Research to Practice:**

This analysis supports several near-term actions: 1. Continue development of a standardized center-level mental health services index (e.g., psychologist/psychiatrist staffing, routine screening for depression/PTSD/substance use, referral pathways, regular follow-up, tele-mental health availability, etc.); 2. merge the index with the BMS national database to build proximity-weighted access maps for post-burn mental health care to prioritize jurisdictions with the largest need-to-supply shortfalls; 3. integrate these metrics into verification and quality improvement screenings with follow-up benchmarks. Collectively, these steps guide equitable resource allocation and follow up care.

**Funding for the study:**

N/A.

